# Afebrile tuberculous prostatic abscess with rectal fistula after intravesical Bacillus Calmette‐Guérin immunotherapy

**DOI:** 10.1002/iju5.12814

**Published:** 2024-11-22

**Authors:** Tatsuhiro Sawada, Ayaka Igarashi, Seiji Arai, Akira Ohtsu, Yuji Fujizuka, Shun Nakazawa, Yoshitaka Sekine, Hidekazu Koike, Yosuke Furuya, Kazuhiro Suzuki

**Affiliations:** ^1^ Department of Urology Gunma University Hospital Maebashi Gunma Japan; ^2^ Department of Urology Kurosawa Hospital Takasaki Gunma Japan

**Keywords:** Abscess, BCG, bladder cancer, prostate, rectal fistula

## Abstract

**Introduction:**

Intravesical Bacillus Calmette‐Guérin immunotherapy is generally a safe treatment for non‐muscle‐invasive bladder cancer but sometimes causes complications.

**Case presentation:**

The patient was an 80‐year‐old man who had undergone Bacillus Calmette‐Guérin immunotherapy for non‐muscle‐invasive bladder cancer. Two months later, he developed an irregular pelvic mass surrounding the prostate and rectum with no fever. A colonoscopy showed purulent mucus discharge in the lower rectum, and a CT‐guided needle biopsy revealed epithelioid granuloma containing Langhans giant cells. Although acid‐fast bacteria culture and PCR of biopsy samples were negative, he was clinically diagnosed with Bacillus Calmette‐Guérin‐related tuberculous prostatic abscess spreading to the rectum. After receiving combined antitubercular drugs for 6 months, his discomfort disappeared with almost complete shrinkage of the prostatic abscess.

**Conclusion:**

Tuberculous prostatic abscess is a rare complication associated with Bacillus Calmette‐Guérin immunotherapy and sometimes induces rectal fistula. Conservative treatment with antitubercular drugs is efficient and safe for treatment of tuberculous prostatic abscess.

**Keynote message**

Intravesical BCG immunotherapy occasionally causes the rare complication of afebrile tuberculous prostatic abscess with rectal fistula. Conservative treatment with combined antitubercular drugs is efficient and safe for this complication.

**Abbreviations & Acronyms**
BCG: Bacillus Calmette‐Guérin
CT: computed tomography
MRI: magnetic resonance imaging
NMIBC: non‐muscle‐invasive bladder cancer
TURBT: transurethral resection for bladder tumor

## Introduction

Intravesical BCG immunotherapy is used for treatment of NMIBC after TURBT.^1^ The therapy is generally safe but sometimes causes complications.

## Case presentation

An 80‐year‐old man underwent TURBT, and the pathological examination revealed high‐grade NMIBC with carcinoma *in situ*. He subsequently received weekly alternating intravesical BCG/epirubicin infusion therapies for 8 weeks, consisting of four infusions of BCG (Tokyo strain; 40 mg) and four infusions of epirubicin (40 mg), with no traumatic catheterizations. However, at 2 months after treatment completion, he experienced discomfort in his lower abdomen and slightly painful defecation. He initially received oral antibiotics, but the symptoms did not improve and instead gradually worsened. Therefore, he was referred to our hospital.

**Fig. 1:**
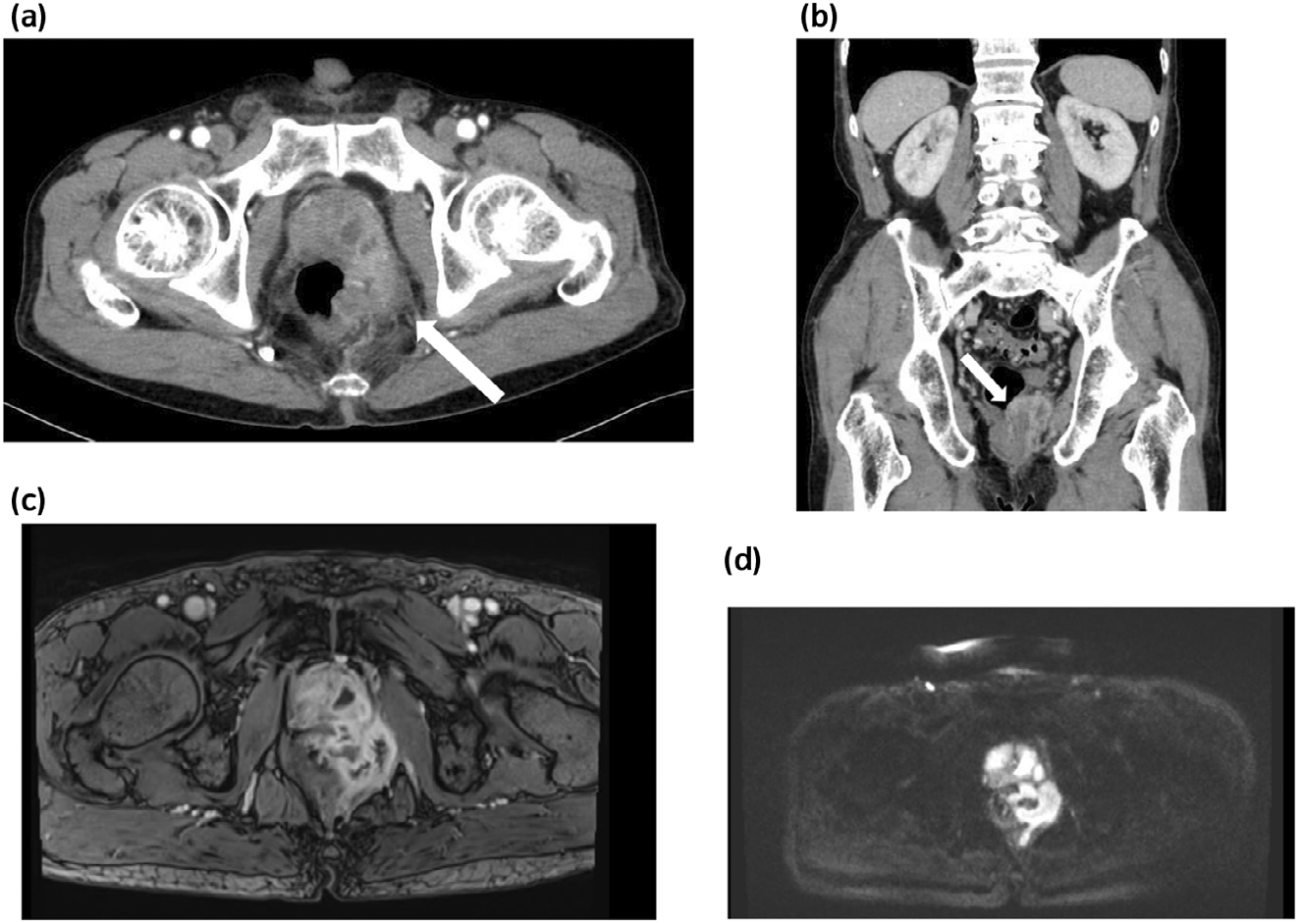
CT and MRI images of the pelvic mass. (a, b) The CT images showed mixed contrast enhancement and low‐density areas in the left prostate lobe, surrounding area and rectum (white arrows; a, axial view; b, sagittal view). (c, d) The MRI images showed contrast enhancement (c, T1‐weighted image) and reduced diffusion (d, diffusion‐weighted image) in the same area.

**Fig. 2:**
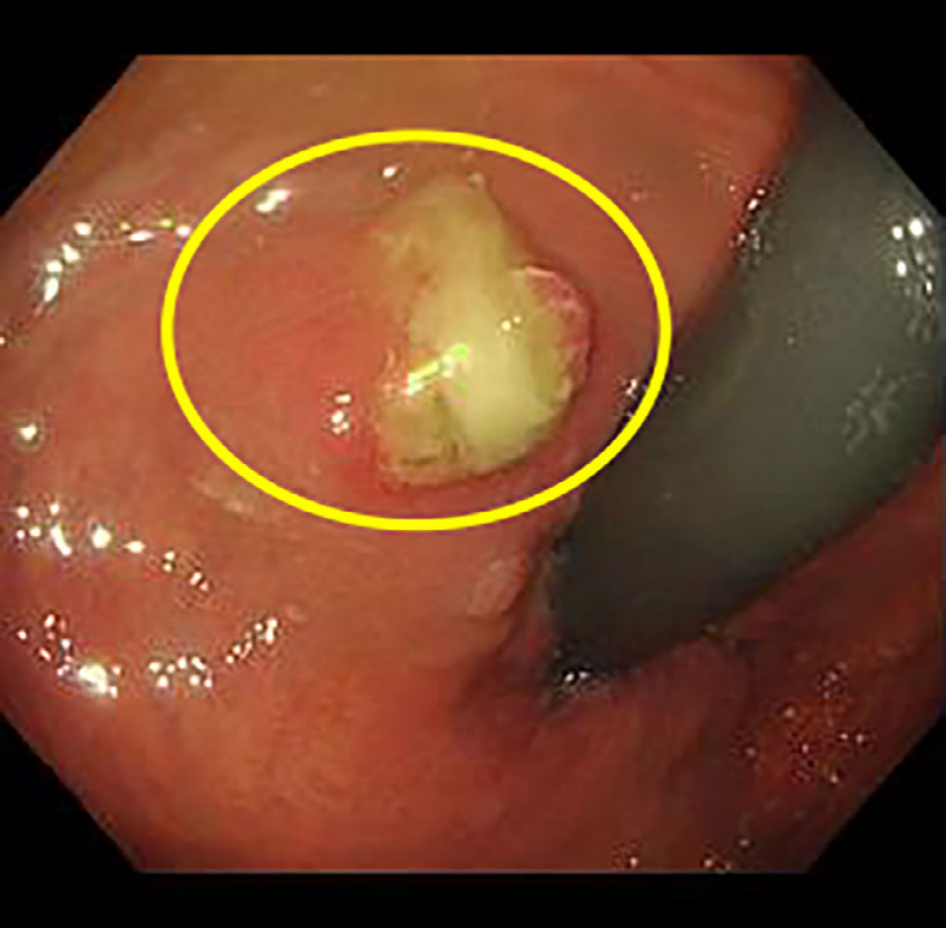
Colonoscope image. The colonoscopy revealed a mucous membrane bulge and purulent mucus discharge in the lower rectum (yellow circle).

**Fig. 3:**
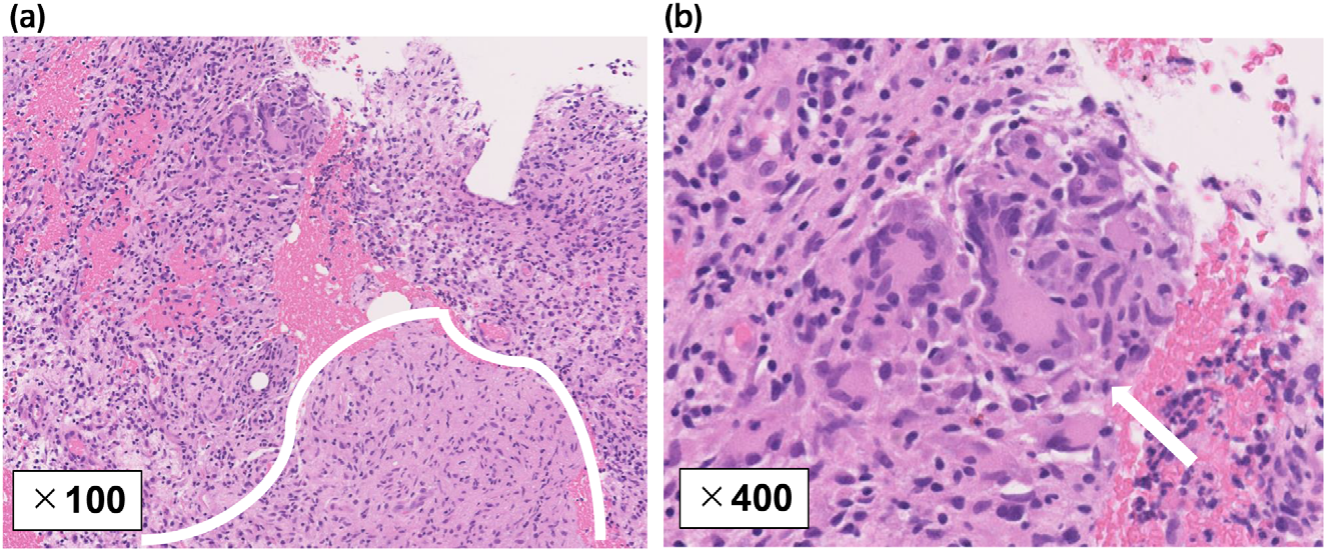
Histopathological findings for the needle biopsy. (a, b) Hematoxylin and eosin staining showed an epithelioid granuloma area (surrounded by a white line) containing Langhans giant cells (white arrow) and infiltration of inflammatory cells (a, ×100 magnification; b, ×400 magnification).

**Fig. 4:**
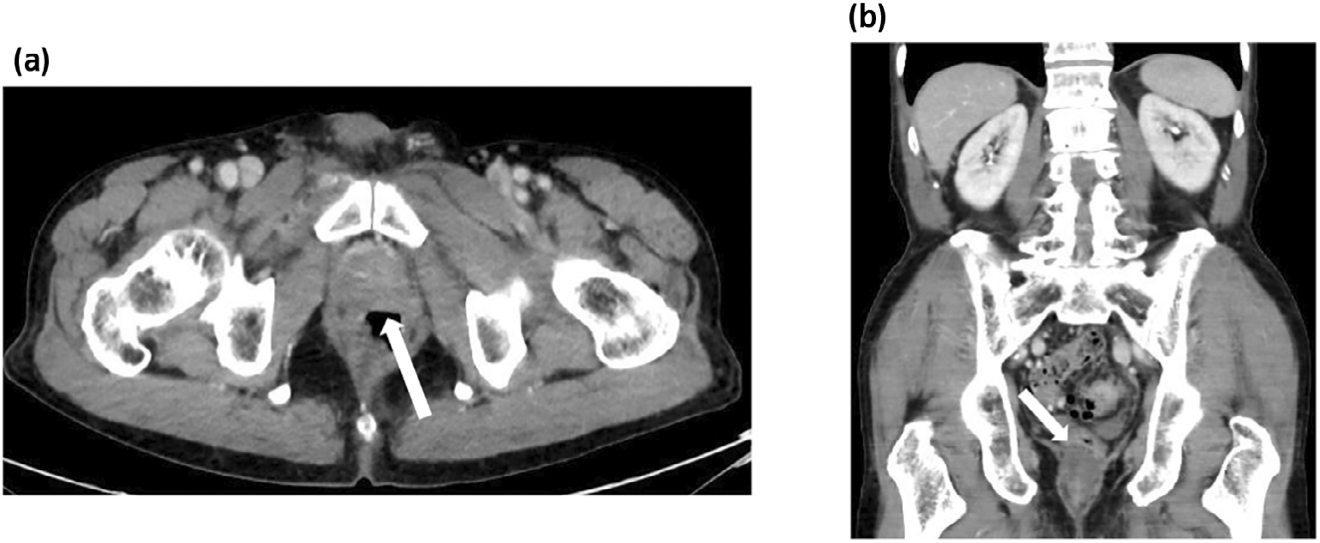
CT images after completion of treatment with combined antitubercular drugs. (a, b) The tuberculous prostatic abscess had almost completely disappeared after completion of the treatment with combined antitubercular drugs (white arrows; a, axial view; b, sagittal view).

He had no fever or apparent urinary symptoms. He also had no specific comorbidities including diabetes mellites. His blood biochemical findings were as follows: white blood cell count (WBC), 5400/μL; C‐reactive protein (CRP), 1.1 mg/dL; prostate‐specific antigen, 3.39 ng/mL. Urinary sediment analysis showed no significant findings. Bacterial culture, acid‐fast bacteria (AFB) culture, and AFB PCR in the urine were negative. A digital rectal examination detected a slightly hard and tender nodule on the left side of the rectum. Contrast‐enhanced CT revealed an incidental gallbladder tumor and an irregular pelvic mass, suggesting either bladder cancer invasion or abscess in the prostate and rectum (Fig. 1a,b). MRI showed contrast enhancement and reduced diffusion in the same area (Fig. 1c,d). A cystoscope examination indicated a small recurrent papillary bladder tumor (Fig. S1). A colonoscopy revealed a mucous membrane bulge and purulent mucus discharge in the lower rectum (Fig. 2), and a biopsy showed inflammatory granulation with no malignant findings. We subsequently performed a CT‐guided needle biopsy (Fig. S2), and the pathological examination revealed epithelioid granuloma containing Langhans giant cells (Fig. 3). Thus, he was clinically diagnosed with small recurrent bladder cancer, gallbladder tumor, and BCG‐related tuberculous prostatic abscess spreading to the rectum, although AFB culture and PCR of the biopsy samples were negative. After a shared process involving the patient and hepatobiliary and pancreatic physicians, we started BCG abscess treatment with isoniazid (300 mg/day), rifampicin (600 mg/day), and ethambutol (1000 mg/day). At 1 month after treatment initiation, the painful defecation and discomfort in the lower abdomen were relieved, and he underwent TURBT for high‐grade NMIBC and staging laparoscopy for the gallbladder tumor (cholecystectomy was initially planned), for which the pathological examination revealed gallbladder cancer with peritoneal dissemination. The patient subsequently received isoniazid, rifampicin, and ethambutol for an additional 1 month and isoniazid and rifampicin therapies for an additional 4 months simultaneously with chemotherapy for the gallbladder cancer. After treatment completion, the tuberculous prostatic abscess had almost completely disappeared (Fig. 4), and the gallbladder cancer was stable.

## Discussion

Intravesical BCG immunotherapy can be used to treat NMIBC, especially in patients with carcinoma *in situ*.^1^ The therapy is generally safe but can cause several complications. A large observational study found infrequent complications of the urinary tract, including granulomatous prostatitis, epididymitis, ureteral obstruction, contracted bladder, and renal abscesses (all ≤1%).^2^ Remarkably, pathological examinations revealed that almost 80% of bladder cancer patients had pathological granulomatous prostatitis in their radical cystoprostatectomy specimens after intravesical BCG treatment.^3^, ^4^ These observations suggest that intravesical BCG treatment causes granulomatous prostatitis in the majority of patients, but only a few patients develop clinically symptomatic granulomatous prostatitis.

Tuberculous prostatic abscess is a rare complication of intravesical BCG immunotherapy and sometimes induces rectal fistula.^5^, ^6^, ^7^, ^8^, ^9^ Thus far, only five cases of BCG‐related tuberculous prostatic abscess have been reported,^5^, ^6^, ^7^, ^8^, ^9^ and all cases with CT images predominantly showed abscess formation in the peripheral zone of the prostate. Notably, pathological granulomatous prostatitis also predominantly or exclusively occurs in the peripheral zone of the prostate, probably due to the microanatomical distribution of the prostate duct,^4^ implying that aggravation of granulomatous prostatitis leads to tuberculous prostatic abscess development, although the specific causal conditions remain unknown. Furthermore, as the prostatic abscess in the peripheral zone worsens, the abscess may rupture the prostatic capsule, spread into the periprostatic and perirectal areas, and finally rupture the rectal wall, forming a rectal fistula.

Bacterial prostatic abscess usually develops in immunocompromised patients, including diabetic patients, due to acute bacterial prostatitis.^10^ Clinical symptoms are typically apparent, including lower urinary tract irritation symptoms in most cases, fever in up to 72%, and perineal pain in 20%.^10^ Intriguingly, all five patients with BCG‐related tuberculous prostatic abscess had continuous lower urinary tract symptoms or pain, but three of the five patients had no fever before diagnosis.^5^, ^6^, ^7^, ^8^, ^9^ These findings suggest distinct pathophysiological features of BCG‐related tuberculous prostatic abscess compared with bacterial prostatic abscess, wherein bacteremia and systemic infection quickly occur. Indeed, the present patient had no fever or serum WBC elevation and only showed slight serum CRP elevation, suggesting that the absence of systemic inflammation and infection and the formation of the rectal fistula may have avoided worsening of the infection through drainage of the abscess.

Because of its infrequent occurrence, the risk factors for BCG‐related tuberculous prostatic abscess remain unknown. However, one study identified large prostate size as an independent predictor of BCG‐related prostatitis.^11^ Assuming that tuberculous prostatic abscess is a worsened stage of granulomatous prostatitis, urologists should pay attention to clinical symptoms in NMIBC patients with benign prostatic hyperplasia who have received intravesical BCG immunotherapy. Notably, the prostate volume in our patient was 32 mL, suggesting that he may have had this risk factor for BCG‐related tuberculous prostatic abscess. Furthermore, the patient simultaneously suffered from double cancer, including disseminated gallbladder cancer, which may have influenced his immunity against BCG, although most patients with solid tumors are not significantly immunocompromised relative to patients with hematologic malignancies.^12^ Conversely, age might impact our patient's BCG‐related prostatic abscess development, whereas several retrospective studies showed that the toxicity of intravesical BCG therapy was not associated with age.^13^, ^14^ Finally, our patient received sequential intravesical BCG/epirubicin therapy, which might also affect the prostatic abscess formation. However, the previous report demonstrated that the sequential intravesical BCG/epirubicin therapy did not increase local and systemic toxicities compared with BCG monotherapy,^15^ suggesting that the sequential treatment was not associated with BCG‐related tuberculous prostatic abscess formation.

The antitubercular agents that can be used against BCG include isoniazid, rifampicin, and ethambutol, although BCG strains are notably insensitive to pyrazinamide.^16^, ^17^ All six cases of BCG‐related tuberculous prostatic abscess (including the present case) were successfully treated with combined antitubercular drugs with or without drainage surgery. These results indicate that conservative treatment with antitubercular drugs is efficient and safe for treatment of tuberculous prostatic abscess.

## Author contributions

Tatsuhiro Sawada: Conceptualization; data curation; writing – review and editing; visualization; investigation. Ayaka Igarashi: Conceptualization; writing – original draft; writing – review and editing; investigation; data curation. Seiji Arai: Writing – original draft; writing – review and editing; visualization; validation; supervision; resources; funding acquisition; conceptualization; investigation; data curation; project administration. Akira Ohtsu: Writing – review and editing; data curation. Yuji Fujizuka: Data curation; writing – review and editing. Shun Nakazawa: Data curation; writing – review and editing. Yoshitaka Sekine: Data curation; writing – review and editing. Hidekazu Koike: Data curation; writing – review and editing. Yosuke Furuya: Data curation; writing – review and editing. Kazuhiro Suzuki: Data curation; writing – review and editing.

## Conflict of interest

The authors declare that they have no competing interests.

## Approval of the research protocol by an Institutional Reviewer Board

Not applicable.

## Informed consent

Written informed consent for the publication of this case report was obtained from the patient.

## Registry and the Registration No. of the study/trial

Not applicable.

## Funding

The authors have no funding to declare for this article.

## Supporting information


**Figure S1.** A cystoscope revealed a small recurrent papillary bladder tumor (yellow circle).
**Figure S2.** A CT‐guided needle biopsy was obtained via the patient's buttocks.
