# Supplementary figures and images for: Afebrile tuberculous prostatic abscess with rectal fistula after intravesical Bacillus Calmette‐Guérin immunotherapy
Source: IJU Case Rep. 2024 Nov 22;8(1):69–72. doi: 10.1002/iju5.12814 (PMC11693102; doi:10.1002/iju5.12814)

## Slide 1
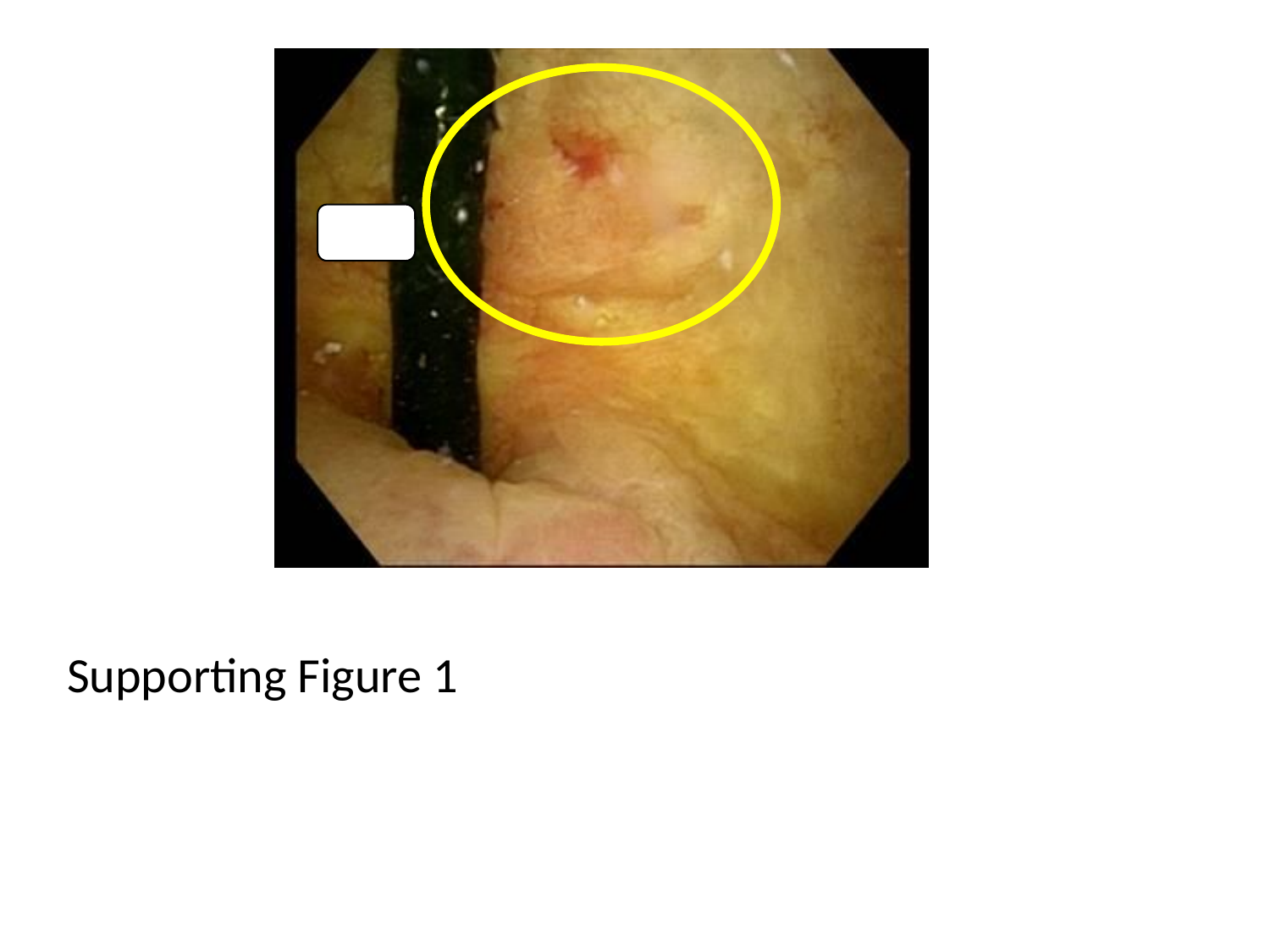

Supporting Figure 1

## Slide 2
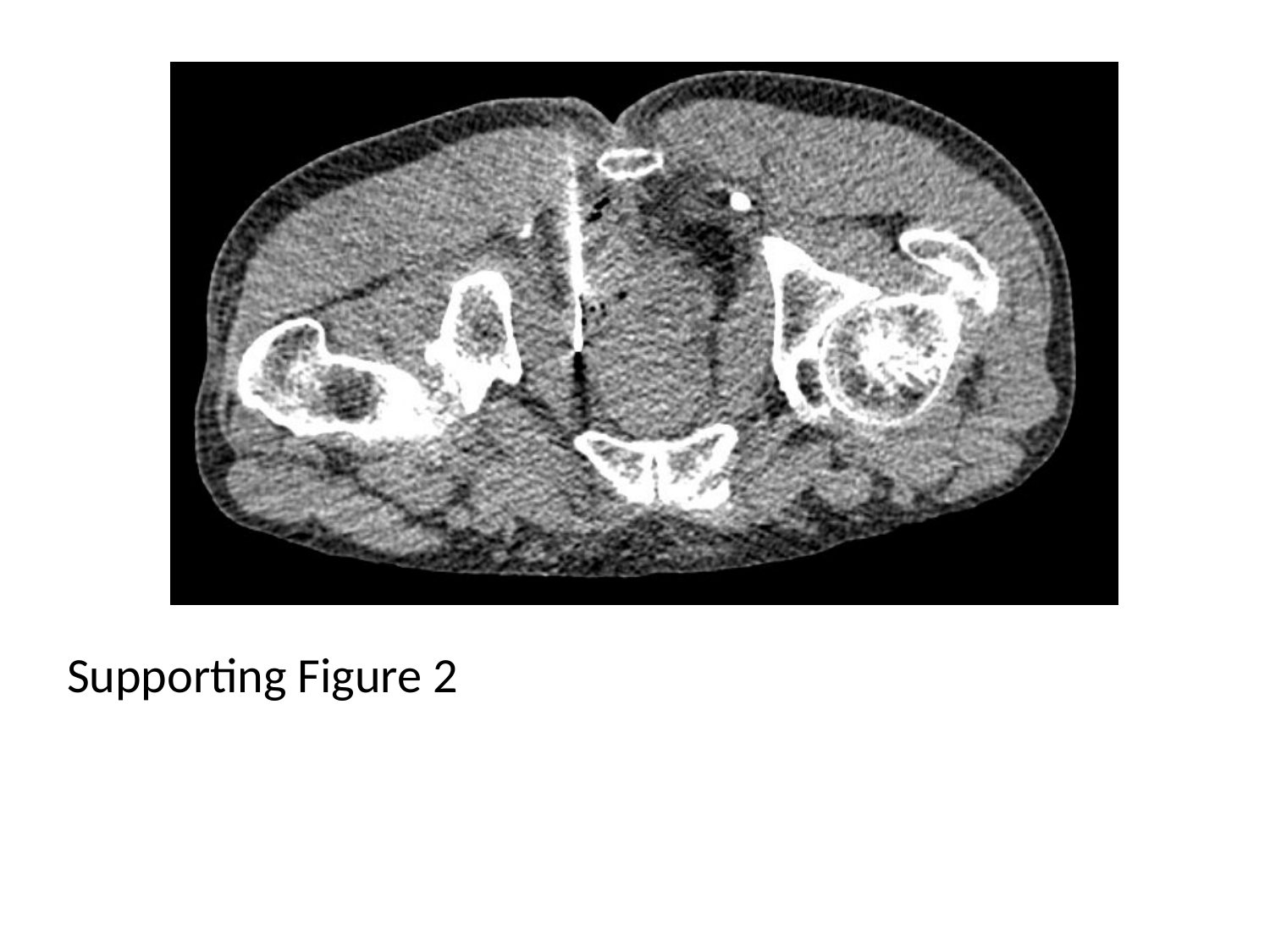

Supporting Figure 2

Supplement: Supplementary file 1 — Figure S1. A cystoscope revealed a small recurrent papillary bladder tumor (yellow circle). Figure S2. A CT‐guided needle biopsy was obtained via the patient's buttocks. [file IJU5-8-69-s001.pptx]
